# The Mnemonic Tuning for Contamination: A Replication and Extension Study Using More Ecologically Valid Stimuli

**DOI:** 10.1177/1474704920946234

**Published:** 2021-03-23

**Authors:** Natália L. Fernandes, Josefa N. S. Pandeirada, James S. Nairne

**Affiliations:** 1 William James Center for Research, University of Aveiro, Portugal; 2 Center for Health Technology and Services Research, University of Aveiro, Portugal; 3 Department of Psychological Sciences, Purdue University, West Lafayette, IN, USA

**Keywords:** adaptive memory, contamination, replication, photographs, objects

## Abstract

To face threats posed by pathogens, natural selection designed the Behavioral Immune System, which orchestrates several responses aimed to prevent contact with pathogens. Memory seems to augment this system. Using line drawings of objects, previous studies found that objects described as having been touched by sick people were better remembered than those described as having been touched by healthy people. The current work was designed to replicate and extend these initial studies using more ecologically-valid stimuli—photographs of real objects being held by hands. These photographs were shown along with descriptors (Experiment 1a) or faces (Experiment 1b) denoting the health status of the person whose hands were holding the objects. Experiments 2 and 3 used, as cues of contamination, dirty hands covered with a substance described as being vomit and diarrhea, respectively. Experiment 3 also investigated the need for a fitness-relevant context for the mnemonic effect to occur. In all experiments, stimuli were presented individually on the screen with the “contamination cue.” During encoding participants had to identify whether each object had been touched by a sick or a healthy person. The results of the final surprise free recall tasks replicated those previously reported: performance was enhanced for objects encoded as potential sources of contamination. Furthermore, the results of the last study reinstate the importance of fitness-relevance for the effect to occur. These results establish the generality of the contamination effect previously found, now using more ecologically-valid stimuli.

Throughout evolution, organisms have been exposed to the challenges imposed by pathogens, favoring the selection of distinct strategies to cope with such life-threatening microorganisms. Among those strategies, probably the best well-known is the biological immune system which detects and eliminates invasive pathogens through the combined efforts of the innate and adaptive immune arms (Parham, 2014; Sompayrac, 2016). A different constellation of mechanisms embodying the “behavioral immune system” (BIS) has been postulated to also play a critical role in defending us against disease-causing microorganisms both in humans and in a wide range of other animal species (Curtis, 2014; Schaller, 2006; Schaller & Duncan, 2007). This system offers unique adaptive benefits by minimizing the exposure to harmful pathogens, thereby preventing the acquisition and transmission of infection in the first place (Ackerman et al., 2018; Schaller & Park, 2011). In humans, when facing environmental cues connoting infection risk, the BIS prompts a cluster of functionally-coordinated psychological processes (i.e., affective, cognitive, and behavioral processes). That is, people feel disgusted by, allocate preferential attention to, and avoid close contact with potential sources of disease, such as conspecifics manifesting signs of infection (Ackerman et al., 2009; Curtis et al., 2004, 2011; Oaten et al., 2009; Schaller & Park, 2011; Tybur & Lieberman, 2016).

Evolutionary psychologists have been proposing that our memory was crafted to solve fitness-relevant adaptive problems, particularly those recurrent in human ancestral environments (Nairne, 2010, 2016; Nairne, Pandeirada, & Fernandes, 2017). Evidence has been accumulating showing mnemonic advantages for fitness-related stimuli, such as animates (e.g., Nairne, VanArsdall, & Cogdill, 2017), potential mating partners (e.g., Pandeirada et al., 2017), intra-sexual rivals (e.g., Becker et al., 2005), and threatening stimuli (e.g., Barrett & Broesch, 2012). In addition, as proposed by Nairne and collaborators, our memory seems to be “tuned” to remember potential sources of contamination (Fernandes et al., 2017; Nairne, 2015). Pathogenic microorganisms pose a serious threat to survival and reproduction (Fumagalli et al., 2011) whereby a mnemonic tuning for contamination is likely to have evolved through natural selection: by remembering disease-threats we were more likely to successfully avoid contact with them and optimize our chances of survival (Fernandes et al., 2017). Accordingly, researchers have reported that individuals are more likely to recall and recognize disgust-eliciting stimuli—commonly associated with transmission paths of pathogens—compared to frightening, positive or neutral stimuli (e.g., Chapman et al., 2013; Ferré et al., 2018).

Driven by the idea that contaminating properties of disgusting items can be transferred to neutral items through contact—the “law of contagion” (Rozin & Fallon, 1987), we have been investigating mnemonic tunings for potentially contaminated items. Specifically, we asked whether people would remember neutral objects that had been touched by sick people (potential sources of contamination) better than when the same objects had been touched by healthy people (Fernandes et al., 2017). In our previous studies, line drawings of objects were presented along with verbal (e.g., short descriptors; Experiment 1a and 1b) or visual cues (e.g., face photographs; Experiment 2) to specify whether that object had been touched by a sick or a healthy person. During encoding, participants had to identify whether each object had been touched by a sick or a healthy person to ensure proper encoding. Near-perfect performance in this immediate memory test in all conditions ensured the stimuli were encoded as intended. In a final surprise free recall task, participants recalled significantly more of the objects previously paired with cues of sickness than those paired with cues of health—in other words, participants retained more of the potentially contaminated objects. It is worth noting that previous studies comparing memory performance for disgusting versus non-disgusting items have typically compared different stimuli (e.g., Chapman et al., 2013; Croucher et al., 2011), which invites alternative accounts based on the items themselves. In fact, even when considerable effort is devoted to equating the stimuli, they may still vary along a number of uncontrolled (and potentially relevant to memory) dimensions (Nairne, 2010). In our experiments everyone was asked to remember exactly the same “neutral” stimuli (which precludes item-selection concerns) but their fitness-relevance was manipulated by framing them as potentially contaminated or not (see also Bell & Buchner, 2010). To our knowledge, these studies provided the first empirical evidence of a memory advantage for neutral stimuli that “acquired” the status of potential contaminants through proximity or brief contact with a source of pathogens (sick people).

Fernandes and collaborators’ work has inspired follow-up studies, seeking to build on and extend our understanding of this *contamination effect*. For example, Bonin et al. (2019) also found that contaminated (vs. non-contaminated) items produced better retention and source identification. During encoding, participants were presented with objects paired with sick- or healthy-looking faces, and were asked to report their perceived discomfort in a hypothetical situation of contact with each object (i.e., incidental learning task: Experiment 5a), or to memorize pairs of stimuli consisting of a sick/healthy face and an object (i.e., intentional learning task: Experiment 5b). Free recall performance and source memory for an item’s condition were better for the contaminated items. In their Experiment 4, participants were asked to pay attention to the pairing of the objects with one of two faces; one was the face of a person suffering from a cold and the other was a face of a healthy person. Again, in a final surprise free recall task, objects paired with the drawing of the sick face were remembered better compared to those paired with the healthy face.

Notwithstanding the contribution of the initial studies by Fernandes and collaborators (2017) and those that followed by Bonin et al. (2019), one could argue that the stimuli that were used (line drawings of objects) lack ecological validity. In fact, line-drawings differ in many important respects from photographs of real objects, and these differences are believed to account for some differences in the processing of stimuli. For example, different semantic processes seem to be activated by these two types of stimuli, which potentially influence how people attend to, name, and recognize objects. As noted by Brodeur et al. (2014), using photographs “increases the chances of activating the same neuronal circuits that are activated in daily tasks” (p. 2), mimicking more closely real-life conditions. Thus, demonstrating the contamination effect with such stimuli would provide additional evidence to this phenomenon. Furthermore, replication is foundational to science, lying at the “heart of scientific progress” (Walker et al., 2017, p. 1221), and is needed to establish the generalizability and reliability of an effect (Pashler & Wagenmakers, 2012; Roediger, 2012).

The current work aimed to replicate and extend the demonstration of the contamination effect using more realistic stimuli—photographs of objects being held by hands, rather than using line-drawings. Using the same procedure as Fernandes et al. (2017), we tested the contamination effect for photographs of objects associated with descriptors (Experiment 1a) or faces (Experiment 1b) denoting the health status of the person who contacted with the object (sick or healthy).

Note that in the studies conducted so far, the to-be-remembered object and the contamination cues (descriptors or faces) were arranged side-by-side without a visible direct contact. For the object to acquire the potential for contamination, participants had to imagine the contact or interaction between the object and the person with that face or with the described characteristic. In two additional experiments, the object was presented in direct physical contact with a possible source of contamination—hands holding the object, making the potential spread of contamination to the objects more readily intelligible to participants. In some cases, the hands were clean whereas in others they were covered with a substance (hereafter referred to as dirty hands) that conveyed the potential source of contamination. In Experiment 2, the objects were held by hands covered with a substance described as being vomit; control objects were held by clean hands. This procedure is in line with recent work by Gretz and Huff (2019). In their experiment, participants saw videos of a person interacting with objects placed in different house compartments and were instructed to remember the objects in the scenes.^1^ Importantly, to different groups of participants, the person was described as having a contagious disease (i.e., influenza), a non-contagious disease (i.e., cancer), or no disease (i.e., healthy). In the final free recall task, everyone recalled more touched- than non-touched objects but this difference, as well as the correct source identification (touched vs. non-touched), was higher in the influenza group as compared to the cancer and healthy groups (the latter did not differ). These results are consistent with the proposal of a mnemonic contamination effect when direct contact exists between the source of contamination and the object.

In Experiment 3, similar to what was done in Experiment 3 of Fernandes et al. (2017), we explored the need for fitness-relevance to obtain a contamination effect. This test was done while continuing to explore the direct transmission of pathogens between the hands and the object being held. As in previous studies, everyone was asked to remember exactly the same items; what differed was whether the object had been in contact with a potential source of contamination or not.

## Experiments 1a and 1b

Experiments 1a and 1b aimed to replicate two of the experiments in Fernandes et al. (2017) using object photographs as the to-be-remembered stimuli instead of object line drawings. In Experiment 1a, condition (sick vs. healthy) was manipulated via the presentation of sentences that described a signal or symptom of disease or a neutral characteristic. In Experiment 1b, we used faces as the cue for contamination; some contained signals of an infectious disease and others contained no such signals.

## Method

### Participants

To determine the sample size for these experiments, we averaged the two effect sizes (*d_z_* = 0.515) reported in the two Experiments that used a similar procedure in Fernandes et al. (2017). Using G*Power (Version 3.1.9.7; Faul et al., 2007), we estimated that 36 participants would be needed to obtain such an effect (two-tailed; α of .05 and power of .85). For counterbalancing reasons and for consistency with the previous experiments, the total final sample in each experiment comprised 48 usable participants. Samples were composed of portuguese Caucasian undergraduate students (female = 21, 43.75% in Experiment 1a; female = 36, 75.0% in Experiment 1b) from the University of Aveiro (*M*_age_ = 21.92 years, *SD* = 3.21; M_age_ = 20.72 years, *SD* = 4.20; in Experiments 1a and 1b, respectively).

Data from 11 additional participants were excluded from analysis because they reported expecting the final memory test and for trying to memorize the stimuli (*n* = 5 and *n* = 6; in Experiments 1a and 1b, respectively). One other participant was excluded from Experiment 1a for having low immediate memory performance (<60% correct). Participants were recruited through face-to-face invitation across campus, via online advertising on the EvoCogLab Facebook page (Experiment 1a), or through in-class announcements (Experiment 1b). Participants were awarded with a small gift or received no compensation. All participants gave written informed consent.

### Materials

**Figure 1.**
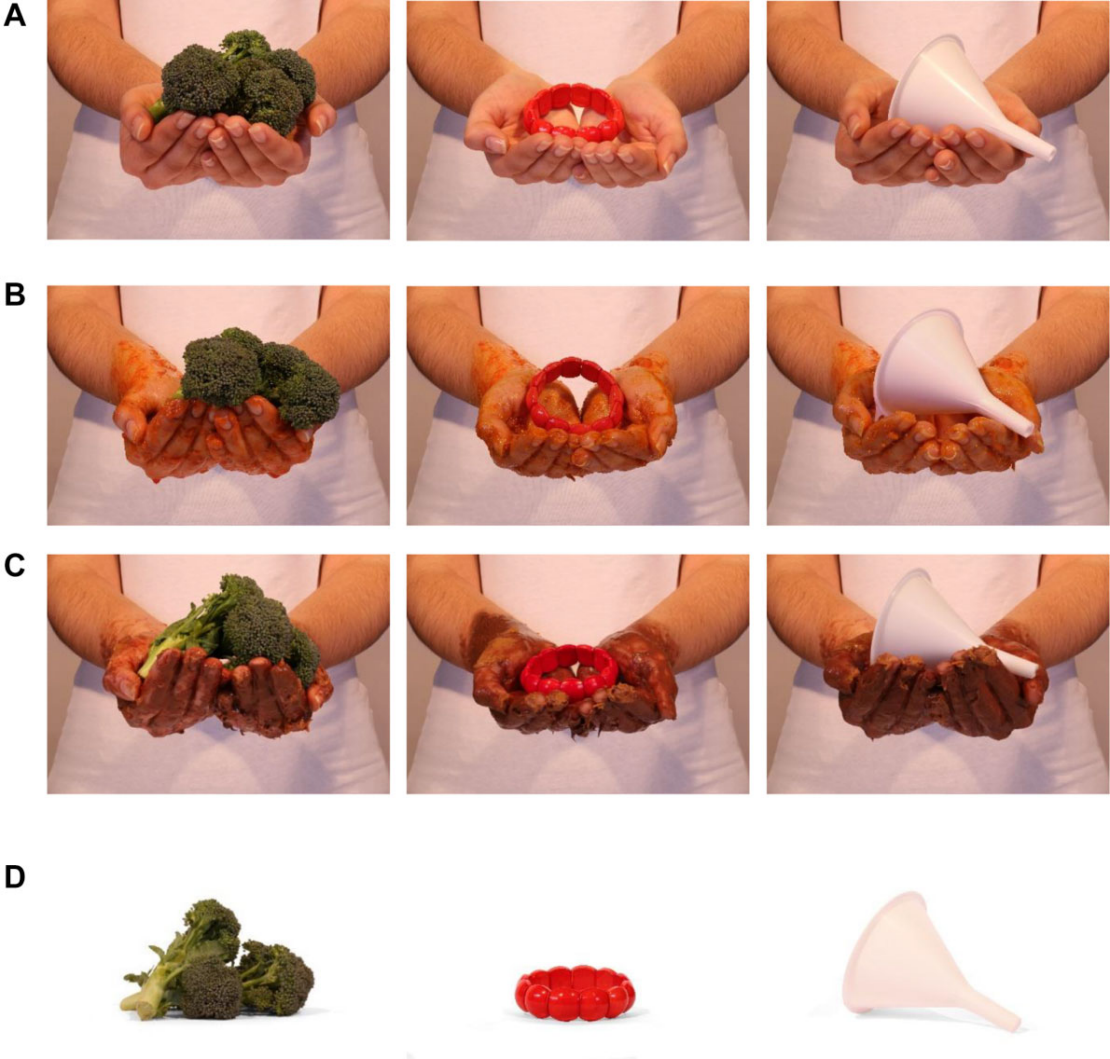
Examples of stimuli used in the different experiments. (A) Used in Experiments 1a and 1b associated with cues (both in the item presentation and the immediate memory phase), and in Experiments 2 and 3 as belonging to healthy people (presentation phase); (B) Used in Experiment 2 as the contaminated items (presentation phase); (C) Used in Experiment 3 during the presentation phase: described as covered with chocolate spread (non-disease context) or as covered with diarrhea (disease context); (D) Used in the immediate memory test of Experiments 2 and 3.

Each stimulus included an object photograph and a descriptor. Twenty-four frontal-view pictures of everyday objects held by clean hands (plus six to be used in practice trials of each experiment) were selected from the Objects-on-Hands Picture Database (for details see Fernandes et al., 2019; see Figure 1 for examples). We selected four and five items from each category (fruits, vegetables, kitchen utensils, office supplies, toys, and women’s accessories) for use in Experiment 1a (total 24 stimuli) and 1b (total 30 stimuli), respectively. According to the Portuguese norming data (Fernandes et al., 2019), the selected items showed high name agreement (i.e., the percentage of participants naming the stimuli with its modal name; %NA = 99.3%, *SD* = 2.0, Experiment 1a; and %NA = 99.1%, *SD* = 1.6, Experiment 1b), and high degree of familiarity (i.e., the degree to which the stimulus is familiar to participants, rated on a 1–5 scale; *M* = 4.82, *SD* = 0.18, Experiment 1a; *M* = 4.80, *SD* = 0.18, Experiment 1b). The objects were then divided in two sets with identical name agreement and familiarity (all *t*s(22) < |1|), and then presented in the sick and healthy conditions in a counterbalanced manner across participants. Each of these sets also contained the same number of items from each object category.

Regarding the cue for contamination, for Experiment 1a, we selected eight of the descriptors used in Fernandes et al. (2017); half described signs and/or symptoms of a sick person (e.g., person with a high fever) and the other half corresponded to neutral characteristics of a person (e.g., person with brown hair). No significant length differences occurred between the sick and healthy descriptors, *t*(6) < |1|. For each participant, the object-descriptor dyads were determined randomly.

For Experiment 1b, we used the thirty female face stimuli described in Fernandes et al. (2017). Each participant saw each face either in its manipulated form (e.g., face containing signs of conjunctivitis, eczema or herpes^2^) or in its normal state (healthy). Participants were not given any verbal description (e.g., “sick” or “healthy”) nor were they informed about the type of illness suffered by each person. The face-object pairings during encoding were randomly determined for each participant.

### Procedure

The procedure was identical to that employed by Fernandes et al. (2017). A single-factor within-subject design was used: each participant saw objects described as having been touched by sick or by healthy people. Instructions used in the current Experiment 1a mimicked those used in Experiment 1 of that work, and those used in Experiment 1b were the same as in Experiment 2. In both experiments, participants were told they would have to remember objects that had been touched by people who were infected with a disease or by healthy people. They were informed that, during the experiment, they would see pictures of objects with a short description / face of the person who had interacted with each object; that information would provide a clue about whether the person who touched the item was sick or healthy and that they would have to remember that information. They were also told that objects and their corresponding short descriptions / faces would be presented one at a time, in sets of three and that, after each set of three, the objects would appear again and they would be asked to remember whether each was touched by a sick or a healthy person. Participants were also informed about the time available to view each stimulus and to make their decision (5 s in each phase per stimuli). No mention was made of the final free recall test.

The encoding phase included presentation of the stimuli and the immediate memory test just described in the instructions provided to participants. Specifically, during presentation, each object picture was displayed on the screen with a descriptor below it (Experiment 1a) or a face presented above the object picture (Experiment 1b), which gave a clue about whether the item had been touched by a sick or a healthy person. After each third stimulus, the immediate memory task followed in which the just presented three objects were presented again, individually in a new random order, and participants had to identify if each item had been touched by a sick or a healthy person. In Experiment 1a eight sets of three stimuli each were presented (total of 24 stimuli) and in Experiment 1b 10 sets of three stimuli were presented (total of 30 stimuli); in both experiments, six additional stimuli were used in an initial practice phase. An equal number of stimuli per condition was presented in each half of the experiment and condition was counterbalanced across participants ensuring that each object participated the same number of times in each condition.

A 2-min distractor task (even-odd discrimination task of singly presented digits) occurred immediately after encoding. Finally, participants were surprised with a free recall task which asked them to recall as many of the objects shown previously as they could in any order and regardless of the “type of person” previously paired with the object. Responses were written on a recall sheet handed out by the researcher during a 10 min period in Experiment 1a. In Experiment 1b, the free recall task lasted for 5 min and was then followed by a surprise source memory task; for this task, participants went over their recalled objects and indicated whether each had been “touched” by a sick or a healthy person. After completing these tasks participants were asked if they anticipated being asked to recall all of the objects and if they had tried to memorize them for a later test. Participants who responded affirmatively to these questions were excluded, ensuring the incidental encoding nature of the task.

Participants were tested on individual computers in groups of up to 6 (Experiment 1a) or 20 participants (Experiment 1b). Each session lasted approximately 20–30 minutes. The experiment was controlled with the software E-prime 2.0 Professional (Schneider et al., 2002). Analyses were performed using the Statistical Package for Social Sciences (IBM SPSS) version 21. The statistical level of significance was set at *p* < .05 for all analyses. For the memory effects (immediate memory, free recall and source memory) we adopted a one-tailed level of significance, given these were predicted a priori; for the reaction times during the immediate memory task, a two-tailed significance level was adopted given no directional prediction was made. The raw data of these experiments and those that follow are publicly available at evo.psych.purdue.edu.

## Results

### Immediate Memory

In both experiments, performance on the immediate memory task was close to ceiling (see Table 1), with no significant differences obtained between conditions, both *ts*(47) < |1|. This result indicates that participants successfully identified the objects as having been presented with a sick or a healthy descriptor or face. Decisions during this task took approximately 1 s in both conditions in Experiment 1a, *t*(47) < |1|. In Experiment 1b, participants also took about 1 s to make their decisions but, in this case, they were faster at deciding about the sick stimuli than about the healthy stimuli, *t*(47) = −2.53, *p* = .015, *d_z_* = 0.365 (see Table 1).

**Table 1.** Mean (and *SD*) of Proportion Correct Responses in the Immediate Memory Task and of Time Taken to Respond in This Task (ms), for the Sick and Healthy Stimuli in Experiments 1a and 1b, and for the Dirty and Clean Hands in Experiments 2 and 3.

	Immediate Memory Performance		Response Time	
Experiment	Sick/Dirty	Healthy/Clean	Sick/Dirty	Healthy/Clean
Experiment 1a	.95 (.07)	.94 (.09)	1,083 (324)	1,116 (318)
Experiment 1b	.96 (.06)	.95 (.08)	1,193 (283)	1,292 (308)
Experiment 2	.95 (.08)	.95 (.09)	1,048 (342)	1,056 (341)
Experiment 3: disease	.98 (.04)	.96 (.06)	966 (288)	958 (316)
Experiment 3: non-disease	.98 (.04)	.97 (.04)	1,046 (321)	1,022 (302)

### Memory Tasks

**Figure 2.**
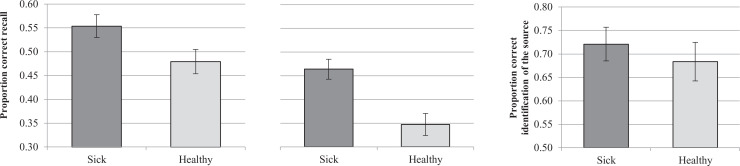
Average proportion of correct free recall for each condition in Experiment 1a (on the left) and 1b (on the center), and of correct source identification in Experiment 1b (on the right). *Note*. Error bars represent standard errors of the mean.

Participants remembered significantly more of the items previously associated with the sick descriptors compared to those previously associated with the healthy descriptors (see Figure 2); this difference reached statistical significance as confirmed by paired-sample *t*-tests at both the subject, *t*(47) = 2.31, *p* = .025, *d_z_* = 0.334,^3^ and item-levels, *t*(23) = 2.40, *p* = .025, *d_z_* = 0.490, in Experiment 1a. The same pattern of results was obtained in Experiment 1b, at the subject, *t*(47) = 4.08, *p* < .001, *d_z_* = 0.589^3^, and item-levels, *t*(29) = 4.96, *p* < .001, *d_z_* = 0.905 (see Figure 2).

Regarding the source memory task in Experiment 1b, one participant exclusively recalled objects from the sick condition and accurately identified the source in all cases; his/her data were not included in the analysis. Nine participants seemed reluctant to guess when unsure of a response and did not provide a source memory response for about 32% (*SD* = 20.6) of their recalled objects. The objects without a source memory response were mainly from the sick condition (57.9%). Because these participants provided a response to the remaining recalled objects, their data were still included. Overall, participants did not differ in their ability to identify the source of the objects that had been previously paired with sick and healthy faces, *t*(46) < |1|^3^ (see Figure 2). Following Fernandes et al. (2017), we analyzed the possibility of a response bias by analyzing the source memory responses to the intrusions. There were relatively few intrusions (*M* = 0.31 per participant), half of which were attributed to the healthy condition, 33.3% to the sick condition, and no source was given for the remaining 16.7% of the intrusions.

### Interim Discussion

These two presented Experiments confirmed that items associated with cues indicative of potential contamination were remembered particularly well as compared to those associated with non-contamination cues. These results replicate those reported by Fernandes et al. (2017) using two types of cues (sentences and faces) now using photos of real objects as the to-be-remembered information. The source memory effect reported in the original study, though, was not replicated, a result we address in the general discussion.

## Experiment 2

In the previously presented Experiments, the potential for contamination had to be imagined by the participants because the object and potential source of contamination (descriptors: Experiment 1a, and faces: Experiment 1b) were presented without visible direct contact. In the next study, objects were shown in direct physical contact with (non-)contamination sources, making the pathogens’ spread from the person to the object more easily attained. Bodily secretions such as vomit serve as a reservoir of pathogens with just 1 ml of vomit from a sick person containing around 10^7^ viral particles (Barker et al., 2001). In Experiment 2, the to-be-remembered objects were presented held either by hands covered with a vomit-looking substance described as belonging to sick people (“sick items”), or by clean hands described as belonging to healthy people (“healthy items”). We expected to replicate previous findings of better memory for the objects that acquired a potential for contamination.

## Method

### Participants

Forty-eight undergraduate students (females = 17; 35.42%) enrolled in an introductory psychology course at Purdue University (USA) consented to participate in the experiment in return for course credits (*M_age_* = 18.92 years, *SD* = 1.10). This sample size corresponds to the one pre-determined for the previous experiments. Data from additional 11 non-native English speakers and 12 participants who suspected the final memory task were excluded. Recruitment was made through the University’s Research Participation System.

### Materials

Twenty-four frontal-view pictures of everyday objects being held by clean hands (non-contaminated items), by hands covered with a vomit-looking pasta sauce (contaminated items), and on their own (plus six of each to be used in practice trials) were selected from the Objects-on-Hands Picture Database (Fernandes et al., 2019; see Figure 1 for examples). According to the American norms, the selected stimuli had high name agreement (%NA = 96.9%, *SD* = 4.6), and high degree of familiarity (*M* = 4.63, *SD* = 0.31; on a scale of 1–5). The images were arranged in two identical sets, with similar name agreement and familiarity, *t*s(22) < |1|. Four counterbalancing versions were created to ensure that each set appeared an equal number of times in its dirty and clean version across participants. Each participant saw each stimulus in only one of these conditions.

### Procedure

Up to four participants were tested in each session in individual workstations; sessions lasted approximately 30 min. The procedure was similar to that used in the previous studies (i.e., an encoding task followed by a distractor and finally by a surprise free recall task). The encoding instructions used in this experiment were as follow:

In this task, you will be asked to remember items that have been touched by different people. Some of these people are sick with a highly contagious disease and have recently thrown up while handling the items, whereas others are healthy people with clean hands. Throughout the experiment, you will see pictures of items being held either by hands covered with vomit or by clean hands. You will need to decide whether the item was touched by a sick or a healthy person and then remember this information for a memory test. (…)

Pictures of objects were presented centrally, first on their own (without cues) during 2 s and then held by the hands (clean or dirty) for 3 s. As before, after each triad, the just three presented objects were again displayed on the screen on their own and participants had to indicate if the object had been touched by a “sick” or a “healthy” person. Participants were given 5 s to decide. This procedure was repeated 8 times (total of 24 stimuli). The distractor task was the same as described in the previous Experiments. The instructions for the final surprise memory task were also as before but now the task lasted for 8 min; the same suspicion control question was also made at the end. Finally, participants viewed all of the photographs of the objects being held by hands presented during encoding (clean and dirty stimuli) and rated how calm or excited each picture made them feel (i.e., arousal) using a 9-point Likert scale (1 = very calm, relaxed, sleepy, or some other similar feeling; 9 = very excited, jittery, wide-awake, or some other similar feeling). Participants were fully debriefed at the end of the experiment.

## Results

### Immediate Memory

Participants performed at about 95% in both conditions, *t*(47) < |1|, suggesting an effective association of the object to its condition (contamination/no-contamination). Approximately 1 s was taken to identify if the item had been touched by a sick or a healthy person, *t*(47) < |1| (see Table 1).

### Free Recall

**Figure 3.**
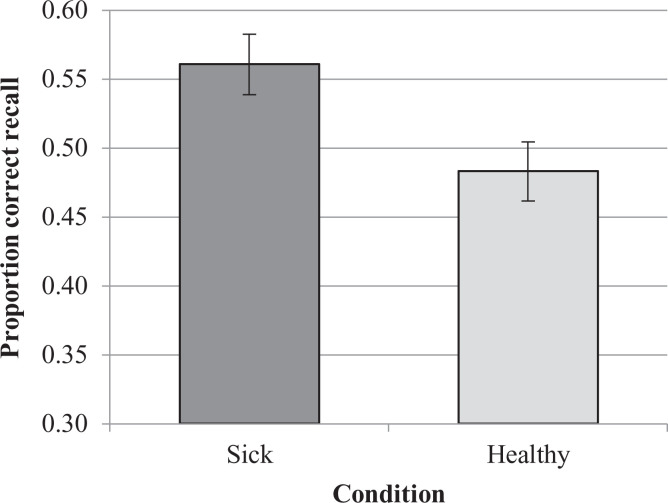
Average proportion of correct free recall for each condition in Experiment 2. *Note*. Error bars represent standard errors of the mean.

Memory performance was significantly better for the “contaminated” items than for the “non-contaminated” items (see Figure 3), at both the subject, *t*(47) = 2.91, *p* = .006, *d_z_* = 0.419^3^, and item levels, *t*(23) = 2.88, *p* = .009, *d_z_* = 0.587.

### Arousal Ratings

Participants reported being significantly more aroused by images of objects held by dirty hands than when the same objects were held by clean hands (*M* = 5.35, *SD* = 1.72 and *M* = 2.46, *SD* = 1.31, respectively), *t*(47) = 8.03, *p* < .001, *d_z_* = 1.159. To determine whether the recall advantage for the contaminated items was related to the arousal rating, a Pearson correlation was performed. Difference scores were calculated for each participant on recall and arousal rating by subtracting the scores for the contamination condition from those for the non-contamination condition. No significant correlation was found between these two difference scores (*r* = −.03, *p* = .818), suggesting that the mnemonic tuning for contamination observed in this Experiment is unrelated to arousal.

### Interim Discussion

Results from Experiment 2 confirmed, once again, our main hypothesis: the objects described as potential sources of contamination were better remembered than those that carried a lower risk of contamination. This demonstration was now obtained in a manipulation in which objects were presented in close contact with the source of contamination.

## Experiment 3

The purpose of Experiment 3 was twofold. Firstly, we sought to replicate the findings of Experiment 2 using another vehicle of contamination: diarrhea. Feces are another potential source of infection; feces contain at least 20 known bacterial, viral, and protozoan pathogens that pose a high risk of infection; 1 g of feces contains an estimated 10^12^ viral particles (Barker et al., 2001). Speculating that the BIS must be adaptable to different sources of pathogens, we expected to replicate the results of the last-reported experiment. The procedure of the previous experiment was followed here but now, in the contamination condition, objects were presented on hands covered with a chocolate and peanut-butter spread that looked like diarrhea. Secondly, we wanted to test whether an attribution of fitness-relevance is required to obtain the mnemonic effect for the “dirty items.” Toward that end, two groups of participants took part in this experiment. Importantly, for one of the groups, the substance covering the dirty hands was described as being diarrhea and, for the other group, it was described as being chocolate spread. Thus, memory for the same objects being held by the same hands was tested, but the fitness-relevance of the context (disease vs. non-disease) varied between groups.

## Method

### Participants

Considering this experiment contains one within- and one between-subjects factor, and that we are also looking for an interaction between these factors, using the same criteria used to pre-determine the sample size of the previous experiments (i.e., *d_z_* = 0.515, α of .05, and power of .85), a sample of 66 participants would be required. We opted to run a slightly larger sample to keep consistent with the previous experiments. Eighty undergraduate psychology students (females = 34; 42.5%) from Purdue University (USA) took part in the experiment in exchange for course credits (*M_age_* = 19.60, *SD* = 1.31). Half of the participants (*n* = 40) were assigned to the disease context and the other half to the non-disease context. A further 32 participants were excluded for the following reasons: non-native English speakers (*n* = 18), not indicating their nationality (*n* = 1), expecting the final memory test (*n* = 9), being under the age of 18 (*n* = 1), or having low immediate memory performance (<60% correct, *n* = 3). Written informed consent was granted by all participants. Recruitment was made through the University’s Research Participation System.

### Materials

A total of 24 photos of objects were selected from the Objects-on-Hands Picture Database (Fernandes et al., 2019) for use in the experiment (plus 6 of each for the practice trials). All pictures selected had high name agreement (%NA = 97.90%, *SD* = 5.48) and familiarity scores (*M* = 4.76, *SD* = 0.19; on a scale of 1–5) according to the American norms. The stimuli were comprised of frontal-view pictures of each object being held by clean hands, by hands covered with a mixture of chocolate spread and peanut butter, and on its own (see Figure 1 for examples).

As in Experiment 2, two lists of stimuli were created, one to be assigned to the clean condition and the other to the dirty condition; this assignment was counterbalanced across participants, as was the order of stimulus presentation, yielding four counterbalancing versions for each context (i.e., disease and non-disease contexts). Thus, each object was only presented once to a given participant in one of the conditions (clean or dirty).

### Procedure

The procedure used in this experiment was analogous to that described in Experiment 2, while accommodating the fact that we now manipulated the encoding context between subjects. On arrival at the laboratory, participants were randomly assigned to one of the versions of the experiment and to the disease or non-disease condition. The specific instructions for each context were as follows:

**Initial encoding instructions common to the two groups:** “In this experiment, you will be asked to remember items that have been touched by different people. First you will see a description of each person. Then, items on hands will be presented one at a time, in sets of three. After each set of three, the items will appear again and you will be asked to remember who touched it.”

**Disease Context:** “Zonia has a highly contagious gastrointestinal infection and is having severe and frequent episodes of diarrhea. Sometimes she cannot reach the toilet on time and gets diarrhea on her hands while handling objects. Marin has a newborn child and is having to stay at home to take care of him. Sometimes she cannot help but worry about her child’s safety and is careful to have clean hands while handling objects.

Throughout the experiment, you will see pictures of items being held either by Zonia, whose hands are covered with diarrhea, or by Marin, whose hands are clean. You will need to decide whether the item was touched by Zonia or Marin and then remember this information for a memory test.”

**Non-Disease Context:** “Zonia bought lots of groceries and is having to make cakes and organize the house for a birthday party. Sometimes she cannot find time to clean her hands and has chocolate spread on them while handling objects. Marin has a newborn child and is having to stay at home to take care of him. Sometimes she cannot help but worry about her child’s safety and is careful to have clean hands while handling objects.

Throughout the experiment, you will see pictures of items being held either by Zonia, whose hands are covered with chocolate spread, or by Marin, whose hands are clean. You will need to decide whether the item was touched by Zonia or Marin and then remember this information for a memory test.”

**Final encoding instructions common to the two groups:** “If the person who touched the item was Zonia, press the “Z” key at that time. If the person was Marin, press the “M” key. The hands will not be presented at the moment you have to make this decision, so you will need to remember who touched and manipulated each of the items. After you have entered your responses for each item, a new set of three items will be presented and this sequence of tasks will be repeated.”

The procedure described in Experiment 2 was used. At the end of the experiment, participants also responded to the following questions using a 9-point Likert scale: (1) how calm or excited the items touched by each person (Zonia and Marin) made them feel, (2) how disgusted the items touched by each person made them feel, and (3) how likely would someone be to get sick if s/he touched or interacted with items previously touched by each person. Responses to these questions allow us to explore possible mechanisms underlying mnemonic differences as well as the efficacy of our manipulation. The experiment ended with the debriefing.

Given that a mixed design was used, with encoding context (i.e., disease vs. non-disease context) manipulated between subjects and type of hands (i.e., dirty vs. clean) as a within-subject variable, two-way mixed ANOVAs were carried out. Paired-sample *t*-tests were also conducted separately for each context because we made the a priori prediction of a mnemonic advantage in the disease context but not in the non-disease context.

## Results

### Immediate Memory

Participants performed close to perfect in the immediate memory task, with no main effect of type of hands, *F*(1,78) = 2.50, *MSE* = 0.003, *p* = .118, no main effect of context, nor interaction between variables, both *Fs*(1,78) < 1 (see Table 1). Participants took about 1 s to decide who had touched each item. Again, none of the main effects nor the interaction approached significance, *F*(1, 78) = 1.37, *MSE* = 207,164.773 *p* = .245 for the main effect of context, remaining *Fs*(1, 78) < 1 (see Table 1).

### Free Recall

**Figure 4.**
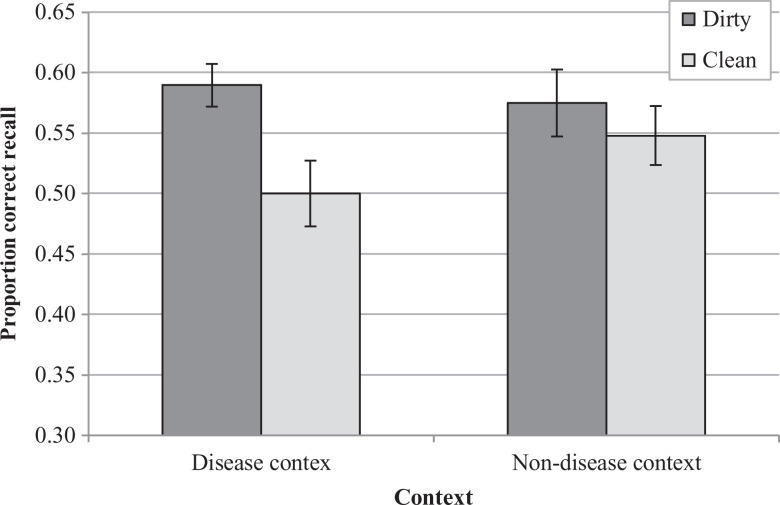
Average proportion of free recall for each condition by participants assigned to the disease and the non-disease context in Experiment 3. *Note*. Error bars represent standard errors of the mean.

A mixed ANOVA revealed a significant main effect of type of hands, *F*(1,78) = 8.18, *MSE* = 0.136, *p* = .005, $\eta_{p}^{2}$ = .095, denoting better memory for the objects when they were held by dirty hands compared to when they were held by clean hands. Neither the main effect of context, *F*(1, 78) < 1, nor the interaction between the two variables was statistically significant, *F*(1, 78) = 2.35, *MSE* = 0.039, *p* = .129. Paired-comparisons conducted separately for each context revealed a significant mnemonic advantage for the dirty objects in the disease context but not in the non-disease context. That is, in the disease context, participants remembered more of the items previously in contact with a potential source of contamination (i.e., those presented on hands described as being covered with diarrhea) compared to those previously presented on clean hands; this effect was obtained both in the subject and in the item analyses, *t*(39) = 3.25, *p* = .002, *d_z_* = 0.514^3^, and *t*(23) = 2.46, *p* = .022, *d_z_* = 0.501, respectively. However, in the non-disease context, this same difference did not approach traditional levels of statistical significance in the subject or the item levels, *t*(39) < |1|^3^ and *t*(23) < |1|, respectively (see Figure 4).

### Ratings

Participants subjectively rated themselves as feeling significantly more aroused, disgusted, and at higher risk of contracting a disease when objects were held by dirty hands than by clean hands. The main effect of the context only occurred in the last two dimensions for which significant interactions were also found. Thus, although in both contexts objects on dirty hands were rated as significantly more disgusting and risky, the effect was stronger in the disease context as compared to the non-disease context (see Table 2 for the descriptive data and accompanying statistical results). As in Experiment 2, we calculated the Pearson correlations between the difference scores in each of these measures and the contamination effect in the disease and the non-disease context. No significant correlations were obtained in either context (higher *r* = .086, *p* = .597, for the correlation between the difference score of arousal and that of recall in the non-disease context), suggesting that the mnemonic advantage obtained (namely the contamination effect observed in the disease context) is unlikely related to arousal, disgust, and risk of contracting a disease.

**Table 2.** Mean Ratings (and Standard Deviations) Obtained for Each Context and Each Type of Hand for the Evaluations of Arousal, Disgust and Likelihood of Becoming Sick (Scale Ranged From 1 to 9) in Experiment 3.

	Disease Context		Non-Disease Context		
	Dirty	Clean	Dirty	Clean	Statistical Results
AROUSAL	5.05 (1.63)	3.60 (1.79)	4.75 (1.78)	3.68 (1.87)	Type of hands: *F*(1,78) = 19.71, *MSE* = 63.756, $\eta_{p}^{2}$ = .202*** Context: *F*(1,78) < 1 Interaction: *F*(1,78) < 1
DISGUST	6.58 (2.23)	1.43 (0.98)	5.00 (2.41)	1.43 (1.08)	Type of hands: *F*(1,78) = 244.92, *MSE* = 761.256, $\eta_{p}^{2}$ = .758*** Context: *F*(1,78) = 7.42, *MSE* = 24.806, $\eta_{p}^{2}$ = .087** Interaction: *F*(1,78) = 7.98, *MSE* = 24.806, $\eta_{p}^{2}$ = .093** Disease Context: *t*(39) = 14.00, *d_Z_* = 2.214*** Non-disease Context: *t*(39) = 8.53, *d_Z_* = 1.348***
DISEASE	7.93 (1.53)	2.88 (2.00)	5.10 (1.84)	2.83 (1.60)	Type of hands: *F*(1,78) = 186.89, *MSE* = 536.556, $\eta_{p}^{2}$ = .706*** Context: *F*(1,78) = 25.32, *MSE* = 82.656, $\eta_{p}^{2}$ = .245 *** Interaction: *F*(1,78) = 26.82, *MSE* = 77.006, $\eta_{p}^{2}$ = .256*** Disease Context: *t*(39) = 13.21, *d_Z_* = 2.089*** Non-disease Context: *t*(39) = 6.06, *d_Z_* = 0.956***

**p < .01. ***p < .001.

### Interim Discussion

In this study, participants were required to remember exactly the same objects held by exactly the same hands but in two contexts that differed in fitness-relevance: Whereas in the disease context the dirty hands were described as potential vehicles of pathogens, in the non-disease context, such risk was absent. As predicted, participants assigned to the disease context recalled significantly more contaminated (i.e., dirty hands) than non-contaminated items (i.e., clean hands), whereas no difference between conditions (dirty vs. clean hands) was found in the non-disease context. We should note, however, that in spite of our initial sample determination, we might lack sufficient power to obtain a significant interaction. Additionally, the differences in the ratings obtained at the end of the experiment suggest that our context manipulation was effective. Still, the characteristics of the dirty hands seem to have somewhat activated participants. We will return to this in the general discussion.

## General Discussion

Our first two experiments were designed to increase the ecological validity of previous work by using photos of objects as the to-be-remembered stimuli. As noted by Peeters (2018), “the use of more ecologically valid stimuli significantly increases the odds of experimental findings being generalizable to everyday situations” (p. 1048). Furthermore, we responded to the appeal for replication studies to help build more solid scientific knowledge (Koole & Lakens, 2012). In both experiments, we replicated the results reported by Fernandes et al. (2017) when sentences and faces were used as the cue for contamination but with photos of objects as the to-be-remembered stimuli. Interestingly, we found a larger contamination effect than that reported by Fernandes et al., particularly in Experiment 1b when both the object and the cues to the health status of the person (i.e., faces photographs) were “real” and more ecologically valid (*d_z_* = 0.589 as compared to *d_z_* = 0.421 reported in that study). However, contrary to what was observed by Fernandes et al., we failed to obtain a significant source memory advantage for the “sick” items in the current Experiment 1b. This result is also at odds with the replication of the source memory advantage for contaminated items recently reported by Bonin et al. (2019).

The results obtained in Experiments 2 and 3 extended the contamination effect to a procedure in which objects were presented in direct contact with the source of contamination (dirty hands), again using real photos. Also, in these experiments only a single cue of contamination was presented during the entire task (i.e., dirty hands), as opposed to the previous studies which used multiple cues (e.g., the faces could contain signs of conjunctivitis, herpes, or Sweet syndrome). A single cue was enough to boost memory for the contaminated objects (as compared to the clean ones) as a contamination advantage was replicated (see also Experiment 4 of Bonin et al., 2019, and Gretz & Huff, 2019). Furthermore, in Experiment 3, we tested and confirmed the need for fitness-relevance to obtain a contamination effect (although we possibly lacked sufficient power to detect a significant statistical interaction). This result parallels those reported by Fernandes et al. (2017; Experiment 3) and by Gretz and Huff (2019); in the later case, the difference in recallability of the objects with which the actor interacted in the video was significantly larger in the infectious disease than in the remaining (non-contagious) conditions.

Overall, these results suggest the effect holds when using different encoding tasks (some that call the participants’ attention to the contamination status of the stimuli and others that do not) and in both intentional and incidental learning conditions (although see Experiments 2 and 3 in Bonin et al., 2019). The contamination effect occurs for objects presented as line drawings and in videos. The current Experiments add photos of real objects to this list and introduce a new way of inducing the contamination threat.

It is interesting to note, though, that in Experiment 3 participants recalled about the same percentage of objects touched by the dirty hands in both the disease and no-disease contexts. This result could relate to an evolved response of the BIS, governed by what has been called “the smoke detector principle” (Schaller & Park, 2011). The failure to detect a real threat (a false-negative error) usually has consequences far more costly than the misinterpretation of an innocuous stimulus as noxious (a false-positive error; Nesse, 2005). Living in a group implies frequent interaction with others, resulting in higher exposure to certain pathogens; thus, people must flexibly adjust signal-detection thresholds to ensure risky disease-threats do not go unnoticed (Kurzban & Leary, 2001; Park et al., 2003). Similarly, researchers have been proposing that the BIS tends to be hypervigilant by setting a low threshold for pathogen detection, in that it is triggered heuristically by any deviation from typical morphology and behavior (features unrelated to contagious; e.g., physical disabilities, Park et al., 2003; facial disfigurements; Ackerman et al., 2009). It is possible that our participants assumed that there was some degree of contamination afforded by the hands covered with “chocolate spread.” This suspicion is to some extent confirmed by the ratings provided by the participants on disgust elicited by the stimuli and the estimated likelihood of someone getting sick in case of a future interaction with items touched by each type of hands. Even though items held by hands with diarrhea were clearly assessed as more likely to contaminate others, the obtained evaluation for the hands covered with chocolate was still significantly higher than that provided to the clean hands. Additionally, participants could be disgusted by the behavior of handling objects without washing the hands in the non-disease context, which failed to conform to conventional norms of health and practices of hygiene. Historically, adherence to cultural norms was likely to be an efficient way to prevent the spread of infectious diseases (Murray & Schaller, 2012). Accordingly, objetcsheld by hands covered with chocolate induced significantly more disgust compared to when the same objects were held by clean hands. Note, however, that the participants’ ratings did not quite reach the top values of the scale (e.g., mean ratings of disgust for hands covered with diarrhea was 6.50 compared to a maximum of 9 points), suggesting that the dirty stimuli and/or the context might not have been completely convincing to participants; this aspect should be explored in future studies.

Likewise, participants may be behaving consistently with the law of similarity. According to this law of sympathetic magic, a harmless stimulus resembling something disgusting can acquire the infectious threat value of the disgusting stimulus, summed up by the idea that “appearance is reality” (e.g., if it looks like feces, it must share some of the disgusting properties of feces). This was illustrated in a well-known study wherein people showed reluctance to try a piece of chocolate when it was shaped in the form of dog feces (Rozin et al., 1986).

Females consistently have been found to be more disgust-prone than males (Sparks et al., 2018) and several theoretical explanations for these sex differences have been offered (see Al-Shawaf et al., 2018, for a systematic analysis). Therefore, if disgust were to be a major determinant of the contamination effect, one would expect females to display larger contamination effects. However, a mixed ANOVA combining the data from the different studies with stimulus condition (i.e., contaminated vs. non-contaminated) as a within-subject variable and sex (female vs. male) as a between-subjects variable, revealed a significant main effect of condition, *F*(1, 182) = 38.41, *MSE* = 0.74, *p* < .001, $\eta_{p}^{2}$ = .174, but neither a main effect of sex nor an interaction between the variables, both *Fs*(1, 182) < 1. These results suggest that both male and female participants remembered contaminated items better that non-contaminated items. However, the interpretation of this finding is constrained by some limitations (e.g., unequal distribution of sex across versions).

Empirical support for a mnemonic advantage for contamination is increasing. Studies demonstrating that memory for survival-relevant information, such as threatening or disgusting information, is better than memory for other types of information abound (e.g., Ackerman et al., 2006; Chapman, 2018). This line of work differs from ours in one important aspect: in our studies, participants remembered exactly the same items in all conditions which solves item-selection problems; critically, the fitness-relevance of the stimuli was acquired via contagion with other threatening elements (e.g., a sick person). Such a mnemonic tuning has been proposed to be a key component of the BIS, a motivational system designed to prevent contact with pathogens (e.g., Schaller & Park, 2011) which, ultimately, maximizes the chances for successful survival and reproduction (Fernandes et al., 2017).

In closing, our studies and those that followed have focused mostly on the ultimate cause of the contamination effect leaving the proximate mechanisms underlying such an advantage largely unexplored (see Nairne & Pandeirada, 2016, for an overview of these approaches). Still, some explanatory hypotheses have been proposed. For example, perhaps people have a stronger emotional reaction or allocate more attentional resources to the contaminated items than to the non-contaminated items (Fernandes et al., 2017). Results from Experiments 2 and 3 suggested that the recall advantage for contamination is not related with variables such as arousal and disgust. Even though the investigation of such mechanisms was not the goal of the present work, it is an area of relevance and future studies should devote to such endeavor to help to fully characterize this phenomenon.

## Notes

1 The use of videos is also a more ecologically-valid form of presenting the stimuli as compared to the previous studies that have used drawings of objects. When our study was designed we were unaware of this study. We believe both these studies and the results of the current experiments expand the robustness of the effect to important forms of stimuli presentation.

2 Examples could be provided upon request.

3 Analyses were repeated including the data of participants that were initially excluded due to suspicion of the final memory task. The inclusion of these participants did not change the reported results.
